# Commentary on: “Agency, time, and causality”

**DOI:** 10.3389/fpsyg.2015.00329

**Published:** 2015-03-24

**Authors:** Guido Peeters

**Affiliations:** Faculty of Psychology and Educational Sciences, Center for Social and Cultural Psychology, University of LeuvenLeuven, Belgium

**Keywords:** agency, causality, culture, cognition, language, personhood, cross-cultural comparison

Cross-cultural comparison of subjective concepts requires a common denominator serving as a basis of comparison. For instance color concepts have been compared across cultures by asking informants to divide the optical spectrum into named colors. Unfortunately similar elegant physical parameters are not always available. A case in point may be the cross-cultural comparison of conceptions of causality discussed recently in *Frontiers* by Widlok (2014). A current approach has contrasted “Western” concepts with non-Western “religious” and “magical” concepts. Widlok has questioned this approach and, relying on an exploration of relevant ethnographic observations, he has proposed an alternative approach of causal cognition involving two parameters: (a) the temporal dimension of sequence, and (b) the concept of agency.

One can very well imagine “time” as a basis of cross-cultural comparison as the optical spectrum is. However, “agency” seems quite a different matter. Exploring concepts of agency in the ethnographic literature, Widlok comes upon distinctions such as between “natural” causes versus causes involving “personhood.” However, it may not always be clear how similar distinctions may map particular culture-specific conceptions of causality involving, for instance, magical and spiritual forces. Hence in the following a formal non-physical basis of comparison is proposed.

In order to compare cognition across cultures we should proceed from a universal feature of cognition. A similar feature would be the organization of cognition into entities and relations reflected by the linguistic noun-verb distinction (Bever, 1970). Using entities and relations as basic cognitive units of analysis, cross-cultural research may tie in with studies involving predominantly Western educated subjects reviewed in the theoretical and discussion sections of two articles available by internet (Peeters, 2004; Peeters and Hendrickx, 2007). For instance, if relations are represented as vectors (arrows), more informational weight seems to be attached to origins (arrow-tails) than to terminals (arrow-heads). Being informed that John likes Pat, subjects attribute likableness to John rather than to Pat. In order to be found likable, Pat should like John in turn (Peeters, 1983). This is in line with Widlok's (2014) observation that Westerners locate causality in the agent rather than in the object acted upon. Replicating the related studies with non-Western Dinka might yield opposite results likableness being attributed to Pat rather than to John.

An important feature of relations concerns reflexivity. A relation is “reflexive” when it forms a loop connecting an entity with itself, and it is “non-reflexive” when it connects one entity with another entity. An all-important structural feature of the architecture of cognition seems to boil down to the double possibility to have reflexivity either attended to or ignored. Attending to reflexivity, subjects deal with entities as “self” and “other” (SO-thought), whereas ignoring reflexivity, they deal with them in the third-person, as “he,” “she,” and “it” (3P-thought). One general finding is that SO-thought is a hallmark of cognitive representations that are intuitively associated with personhood. 3P thought marks rather impersonal cognitive representations such as in hard natural sciences. For instance, given the information that John regales someone with cake, an observer may attribute generosity to John only if “someone” is not John himself but another person (SO-thought). However, in order to draw the rather impersonal inference that John has resources enabling to get cake, the observer may ignore whether the gourmand is John himself or someone else (3P-thought). The connection of attention for reflexivity with attributions of personhood may enable to detect hidden homunculi that, according to Widlok (2014), may be concealed in the curtains of mechanical causal explanations. Alternatively, the disregard of reflexivity may reveal quasi-mechanical thinking about the action of, for instance, magical powers that otherwise might be misconceived as intentional actions of quasi-human ghosts. Notice that there may be no one-to-one relationship between SO/3P thought and the use of correspondent linguistic codes in the communication of those thoughts. SO/3P thought is diagnosed indirectly from the ways subjects accomplish particular experimental tasks that are often more complex than the examples presented. It may require some inventiveness to have them adjusted to cross-cultural research that may proceed from hypotheses like the ones below.

## Hypothesis 1: humans are primarily set for SO-thought

Evidence is so far limited to Western data. For instance, in subjective judgments of fairness, explicit 3P-shaped criteria of fairness were found to be overruled by implicit SO-shaped criteria (Peeters, 1987, 1991). In another study (Peeters et al., 2003) participants were instructed to imitate an experimenter who pointed to a picture of himself ignoring the participant's picture also present. From age five on participants pointed to their own pictures neglecting the experimenter's (SO-thought).

## Hypothesis 2: 3P-thought is restricted to specific domains of cognition

In the above imitation experiment some adult participants pointed to the experimenter's picture (3P-thought). Being asked why, they explained that they mimicked the experimenter “exactly.” The term “exact” was not used by subjects using SO-thought. This surprises because pointing to the own picture would be as exact—in the sense of “strictly correct”—as pointing to the experimenter's. However, for Dutch-speaking adults “exact” connotes “hard science” that is a specific cognitive domain marked by 3P-thought.

## Hypothesis 3: 3P-thought involves more intercultural differences than SO-thought

This is a generalization from the observation that 3P-thought is more flexible than SO-thought making that 3P-shaped cognition varies more between and within subjects depending on perspectives taken by the subjects. For instance, 3P-thought seems related to expertise. Musicians presented with particular human-relations problems manifested SO-thought, but musicians manifested also 3P-thought if the problems were framed in a perspective of musical ensemble (Peeters, 2004).

## Hypothesis 4: SO- and 3P-shaped thought transects habitual distinctions between cognitive domains such as between science and religion

The absence of non-religious hard science in particular cultures does not exclude elaborate domains of 3P-shaped knowledge. In agreement with Widlok (2014), the modes of SO- and 3P-thought do not involve a strict separation between religious and non-religious domains. Western subjects deal with “religion” using both SO-thought and 3P-thought depending on whether religion is viewed as a personal attitude or a doctrine (Peeters and Hendrickx, 2002). Western psychological science has generated 3P-shaped concepts of justice as well as SO-shaped motivational concepts that can account for the same data (Peeters, 1987, 1991).

## Conclusion

Although the entity-relation approach belongs to Western experimental social psychology, it fits in with Widlok's exploration of existing ethnography of causality. The distinction between relations' origins and terminals may capture some aspect of Widlok's dimension of sequence, and relations' reflexivity may provide an operationalization of personhood. Hence the entity-relation approach may not only complete ethnographic observation but also bridge the gap between ethnographic and experimental approaches of cognition, which was a remote goal of Widlok's study.

### Conflict of interest statement

The author declares that the research was conducted in the absence of any commercial or financial relationships that could be construed as a potential conflict of interest.
